# Attention-Demanding Cognitive Tasks Worsen Postural Control in Patients With Cervical Dystonia: A Case-Control Study

**DOI:** 10.3389/fneur.2021.666438

**Published:** 2021-04-06

**Authors:** Viola Baione, Gina Ferrazzano, Claudia Celletti, Matteo De Rosa, Daniele Belvisi, Giovanni Fabbrini, Manuela Galli, Filippo Camerota, Antonella Conte

**Affiliations:** ^1^Department of Human Neuroscience, Sapienza University of Rome, Rome, Italy; ^2^Physical Medicine and Rehabilitation Division, Umberto I University Hospital of Rome, Rome, Italy; ^3^Scientific Institute for Research, Hospitalization and Healthcare (IRCCS) Neuromed, Pozzilli, Italy; ^4^Department of Electronics, Information, and Bioengineering, Politecnico di Milano, Milan, Italy

**Keywords:** cervical dystonia, balance, postural control, cognitive-motor interference, Stroop test, executive functions

## Abstract

**Background:** Patients with cervical dystonia (CD) show impaired postural control, balance, and gait, likely due to abnormal head postures and sensorimotor disturbances. However, until now no study has investigated whether attention-demanding activity worsens postural control and balance in CD patients.

**Objective:** To investigate whether patients with CD show cognitive-motor interference (CMI), a specific kind of dual-task interference that occurs during the simultaneous execution of a cognitive and motor task. This information may be useful to determine whether performing activities of daily living worsens postural control and balance in CD patients.

**Methods:** We performed a pilot case-control study. Twenty-two patients affected by CD and 19 healthy controls were enrolled in order to test CMI. Each subject was evaluated during the execution of a cognitive task while postural stability was assessed through a stabilometric platform.

**Results:** CD patients showed impaired postural control compared to healthy controls, with instability increasing with increasing cognitive task complexity. No relationships were found between stabilometric parameters and clinical characteristics of CD.

**Conclusions:** Our hypothesis is that CMI in CD patients derives from deranged network connectivity when activated simultaneously during the performance of two tasks that interfere with each other and “compete” for the same resources within the cognitive system.

## Introduction

Cervical dystonia (CD), now considered a network disorder, is the most frequent adult-onset focal dystonia (1). CD is characterized by sustained contraction of the neck and shoulder muscles, producing abnormal postures and/or twisting movements of the head and neck (2).

Previous studies have demonstrated that CD patients have impaired postural control, balance, gait, and stepping reactions that could be secondary to abnormal head postures and sensorimotor disturbances (3–6) or may be an endophenotypic aspect of the disease (7).

The dual-task paradigm, i.e., the simultaneous performance of motor and cognitive tasks, can assess the extent that cognitive tasks interfere with automatic motor activity, such as balance and gait, i.e., cognitive-motor interference (CMI) (8, 9). One way to test CMI mechanisms consists of the simultaneous execution of cognitive tests during a postural stability assessment (8). Previous studies have analyzed CMI in patients affected by other movement disorders, i.e., Parkinson's disease (10), essential tremor (11), and Huntington's disease (12), highlighting that dual-task performance severely reduces walking ability in these patients, resulting in an increased risk of falls. However, to date no study has evaluated whether performing attention-demanding activity, e.g., talking on the phone while walking, worsens postural control and balance in CD patients.

If the simultaneous performance of two tasks is worse than what would have been obtained performing only one task at a time, it may be speculated that the two tasks interfere with each other and that they “compete” for the same resources within the cognitive system. We hypothesized that cervical dystonia is not only characterized by the balance alterations due to dystonic posture per sè, but is also associated with a reduction in the functional reserve that is needed for brain mechanisms involved in dual-task performance. Evaluating the impact of dual tasking on postural balance in CD patients may represent a promising tool for detecting subtle disability since it may reflect real-life performance better than assessing motor and cognitive abilities separately.

The main aim of this study was therefore to investigate CMI in a group of CD patients using stabilometric assessment with and without the interference of a neuropsychological tool evaluating multiple cognitive domains. To this aim, we analyzed whether stabilometric parameters change over time during the execution of cognitive tasks in CD patients, evaluated their relationship with clinical features, and compared these results with those obtained from a group of sex- and age-matched healthy controls (HCs). Moreover, to exclude the influence of dystonic posture per sè, we investigated the association between CD patterns (torticollis, laterocollis, anterocollis, retrocollis) and stabilometric parameters during the execution of cognitive tasks.

## Materials and Methods

In this pilot study, we consecutively enrolled 22 CD patients who were diagnosed according to published criteria (13) (six males; mean age 61.8 ± 11.4 years) from March 2019 to July 2019 at the movement disorders clinic of the Department of Human Neuroscience, Sapienza University of Rome. We also enrolled 19 sex- and age-matched HCs (9 males; mean age 59.3 ± 8 years). To exclude any confounding effects due to botulinum toxin (BONT) injections, clinical assessment was performed at least 4 months after the last BONT treatment. The study received approval from the local ethics committee on human experimentation and was conducted in accordance with the Declaration of Helsinki. Written informed consent was obtained from all subjects participating in the study (consent for research). Exclusion criteria were the presence of other neurological disorders and peripheral sensory neuropathy, the use of sedative medications, ankle, hip, or knee disorders, lower limb fractures within 6 months, diabetes, vestibular disorders, or severely impaired vision or color-blindness. Subjects with cognitive impairment (Mini-Mental State Examination (MMSE) score <24; Montreal Cognitive Assessment (MOCA) score <26) and/or psychiatric disturbances as evaluated by the Hamilton Depression Rating Scale (HAM-D) and Hamilton Anxiety Rating Scale (HAM-A) were also excluded. All participants were Italian native speakers in order to avoid bias attributable to language in the Stroop test.

Dystonia severity in CD patients was assessed by a neurologist expert in movement disorders using the revised Toronto Western Spasmodic Torticollis Rating Scale (TWSTRS-2) (14). We also evaluated the presence or absence of head tremor and the specific dystonic pattern (i.e., torticollis, laterocollis, retrocollis, anterocollis, or a combination of these).

Balance and postural control were measured in all subjects through stabilometric assessment, which was performed in the same experimental conditions and in a quiet room at the same standardized distance from the Stroop tables. The stabilometric study was conducted using a pressure platform (BTS Bioengineering P-WALK) composed of 2,304 resistive sensors (10 × 10 mm size) with a dimension of 500/4000 × 480 mm. Acquisition frequency was 100 Hz and the pressure range was 30–400 kpa. Subjects were required to maintain a standing posture on a pressure platform for 30 sec with the feet in a fixed position (30° externally rotated with respect to the anterior axis direction). Participants were instructed to stand upright and as still as possible, with their feet together and hands at their sides while looking at a visual target located in front of them, with a distance of approximately 20 cm between the platform and the wall.

Patients underwent two stabilometric assessments. The first evaluation was performed in a resting condition with the eyes open, i.e., with the subject able to use all possible proprioceptive and exteroceptive information. The second stabilometric evaluation was performed during the execution of the Stroop Color and Word Test (SCWT) (15). During the SCWT, subjects were required to read three different tables as fast as possible. Each test lasted 30 sec. Two of these tables represented the “congruous condition” in which participants were required to read names of colors printed in black ink (the “word task” - W) and name different color patches (the “color task” - C). Conversely, in the third table (the “color-word task” - CW), color words were printed in an inconsistent color ink (e.g., the word “black” was printed in green ink). In this incongruent condition, participants were required to name the color of the ink instead of reading the word, performing a less automated task while inhibiting the interference arising from a more automated task (16). For each subject, the number of correct answers for each trial was recorded. The two evaluations were performed at least 1 week apart in a random order. We calculated the sway area, sway length, mean velocity of center of pressure (COP), and sway signal (X, Y time plot of the COP) at rest and during the execution of all Stroop test trials using the SWAY software program (BTS Engineering). COP is commonly used as a sensitive and objective index of postural stability during quiet standing, representing the displacement over time of the weighted average location of the force vector that accommodates the sway of the body (17). The sway area represents the extent of the total area covered by the COP in a given time (18) and sway signal is a parameter detected by the SWAY software program identified by X, Y time plot of the COP, but it is irrelevant for the measurement of balance. We also calculated the length of surface function (LSF) (18), a variable reflective of the energy spent by the subject in order to maintain postural control, and the Romberg index for COP length of sway, which is the length of Sway with the eyes closed divided by the length of Sway with the eyes open (19). A Romberg index score >1 indicated greater instability with closed eyes.

SPSS version 25 for Windows was used for statistical analysis. Separate two-way ANOVAs were performed to evaluate changes in stabilometric variables (sway length, velocity, sway area, LSF) during the stroop test in CD patients and HCs. Two-way ANOVA was also used to evaluate changes in Stroop test performance between patients and HCs. One-way ANOVA was used to analyze differences in the Romberg index between patients and HCs. Spearman's Rho was used to evaluate any relationships between demographic (age, sex) and clinical (disease severity, disease duration, head tremor, dystonic pattern) variables and stabilometric variables during the Stroop test. A univariate linear regression model using stabilometric parameters as dependent variables and dystonic posture as the independent variable was applied to assess the relationship between CD phenotypes and stabilometric parameters during the Stroop test. A *p*-value < 0.05 indicated statistical significance.

## Results

Participant demographic characteristics and stabilometric data are reported in Table 1. All recruited CD patients and HCs completed the study. Two-way ANOVA for the number of correct responses during the Stroop test disclosed worse performance in CD patients as compared with HCs as shown by a lower number of correct responses and a higher number of errors with increasing cognitive task difficulty (number of correct responses: factor TEST: *F* = 311.9, *p* < 0.0001; GROUP: *F* = 6.47, *p* = 0.01; TEST × GROUP interaction: *F* = 3.19, *p* = 0.04; number of errors: TEST: *F* = 8.11, *p* = 0.0001; TEST × GROUP interaction: *F* = 3.07, *p* = 0.05).

**Table 1:** Demographic, clinical and stabilometric data in CD patients and in HCs.

**Variables**	**CD patients**	**HCs**	**p value**
Gender (M/F)	6/16	9/10	0.2
Age (mean± SD, y)	61.8 ± 11.4	59.3 ± 8	0.04
Duration (mean± SD, y)	10.3 ± 9.2	–	–
Head tremor (%)	59%	–	–
SL T0 (mean ± SD, mm)	133.7 ± 45.7	101.8 ± 19.6	0.1
SL T1 (mean ± SD, mm)	132.8 ± 65.4	98 ± 35.6	0.01
SL T2 (mean ± SD, mm)	150 ± 65.1	89.6 ± 16.5	0.0001
SL T3 (mean ± SD, mm)	158 ± 76	99.6 ± 20.4	0.0001
VO T0 (mean ± SD, mm/s)	4.5 ± 1.5	3.3 ± 0.7	0.1
VO T1 (mean ± SD, mm/s)	4.4 ± 2.2	3.2 ± 1.1	0.01
VO T2 (mean ± SD, mm/s)	4.9 ± 2.1	2.9 ± 0.5	0.0001
VO T3 (mean ± SD, mm/s)	5.3 ± 2.5	3.3 ± 0.6	0.0001
SA T0 (mean ± SD, mm^2^)	79.2 ± 104.8	39.3 ± 37.1	0.08
SA T1 (mean ± SD, mm^2^)	113 ± 188.2	37.5 ± 53.5	0.02
SA T2 (mean ± SD, mm^2^)	125.3 ± 147	23.9 ± 25.8	0.0001
SA T3 (mean ± SD, mm^2^)	186 ± 336	34.1 ± 29.7	0.007
LSF T0 (mean ± SD, mm)	4 ± 4.55	3.7 ± 1.9	0.1
LSF T1 (mean ± SD, mm)	7 ± 8.4	6.4 ± 5.6	0.1
LSF T2 (mean ± SD, mm)	6.3 ± 10.7	8.3 ± 7.8	0.7
LSF T3 (mean ± SD, mm)	7 ± 11.4	5 ± 3.6	0.02

*Data are expressed as mean ± standard deviation (SD) and as the percentage of CD patients. T0, baseline evaluation; T1, T2, T3, are the three Stroop test trials evaluations. CD, cervical dystonia; HCs, healthy controls; Y, years; SL, sway length; LSF, length of surface function; SA, sway area; VO, Velocity of Oscillation; F, females; M, males; mm, millimeter; mm/sec, millimeter/second; mm^2^, square millimeter*.

**Figure 1:**
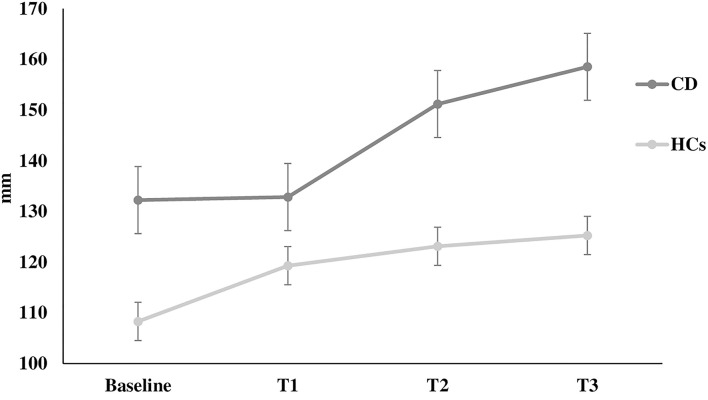
Sway length. Sway length at rest (baseline) and during the execution of all Stroop test trials (T1, T2, T3) in healthy controls and in patients with cervical dystonia. *HCs* healthy controls; *CD* cervical dystonia; *mm* millimeter.

**Figure 2:**
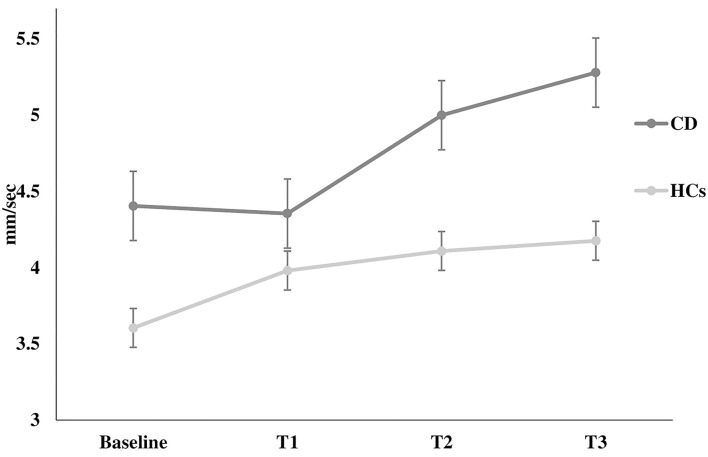
Velocity of oscillation. Velocity of oscillation at rest (baseline) and during the execution of all Stroop test trials (T1, T2, T3) in healthy controls and in patients with cervical dystonia. *HCs* healthy controls; *CD* cervical dystonia; *mm/sec* millimeter/second.

**Figure 3:**
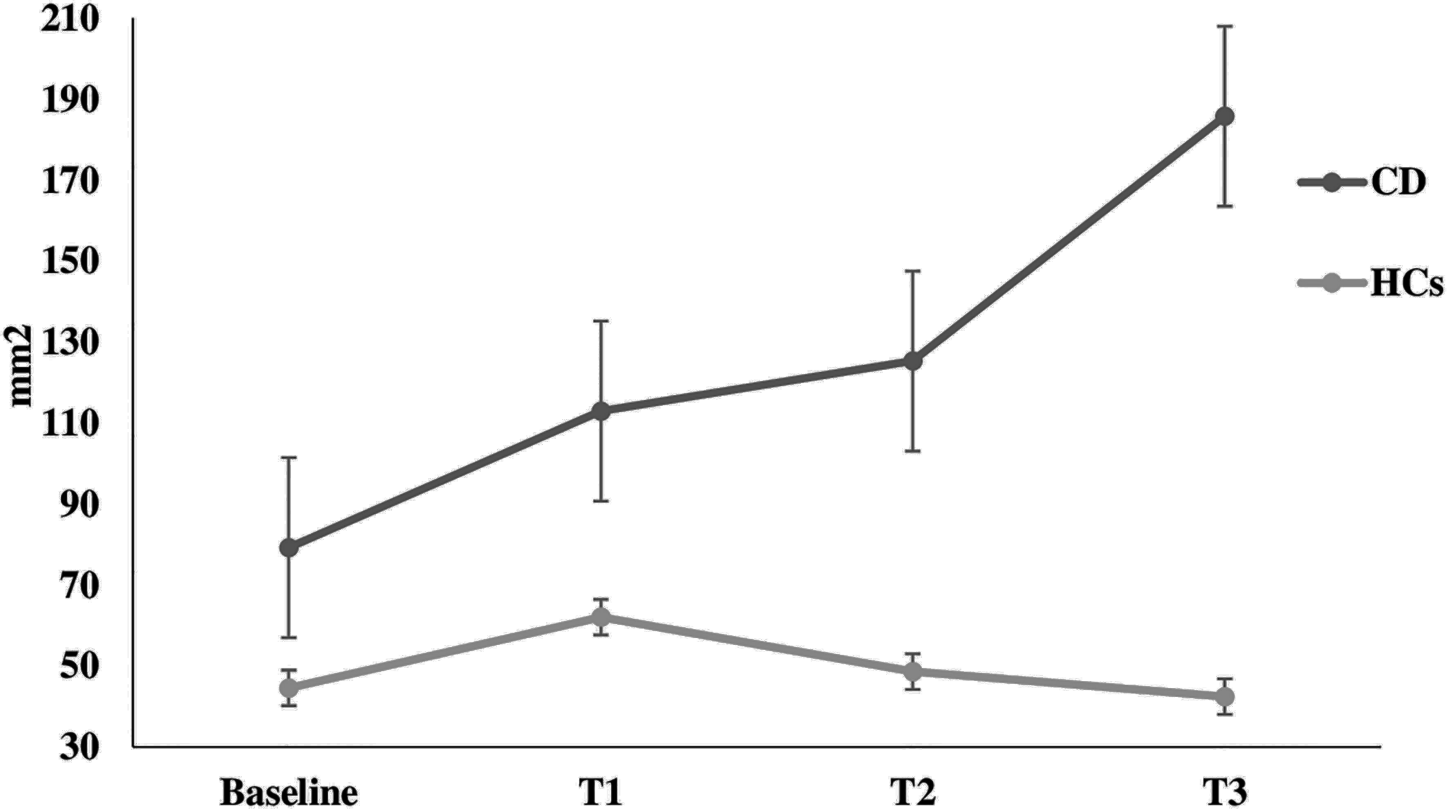
Sway area. Sway area at rest (baseline) and during the execution of all Stroop test trials (T1, T2, T3) in healthy controls and in patients with cervical dystonia. *HCs* healthy controls; *CD* cervical dystonia; *mm*^2^ square millimeter.

The Romberg index did not differ between patients and HCs (*p* > 0.05). However, two-way ANOVA for stabilometric variables during the Stroop test showed that CD patients were less stable than HCs during the Stroop test, with instability increasing with increasing cognitive task complexity (sway length: factor GROUP: *F* = 16.9, *p* < 0.0001, GROUP × TEST interaction: *F* = 3.11, *p* = 0.02; velocity of oscillations: factor GROUP: *F* = 16.3, *p* < 0.0001, GROUP × TEST interaction: *F* = 2.92, *p* = 0.03; sway area: factor GROUP: *F* = 8.22, *p* < 0.001) (Figures 1–3). Stabilometric variables did not differ between patients with (*n* = 13) and without tremor (*n* = 9) (all *p* > 0.05). Spearman correlation analysis disclosed significant correlations between the number of errors made during the third item of the Stroop test and stabilometric variables (number of errors and sway length: Rho = 0.58, *p* = 0.004; number of errors and oscillation velocity: Rho = 0.58, *p* = 0.0045). Finally, no significant relationships were observed between stabilometric variables and disease severity and head tremor as assessed by the TWSTRS-2. Linear regression analysis did not disclose any association between dystonic posture and stabilometric parameters during the Stroop test.

## Discussion

In this study we found that, as compared to HCs, CD patients showed impaired postural control that worsened during the execution of attention-demanding cognitive tasks. Increased balance instability paralleled the complexity of the cognitive task, therefore implying CMI mechanisms. A significant correlation was found between the performance of CD patients during the third trial of the SCWT and stabilometric parameters. Finally, no relationship was found between balance properties and disease severity as measured by the TWSTRS-2 and the presence of head tremor.

All enrolled subjects met the inclusion criteria. Since CD patients and HCs were age-matched, we can exclude the possibility that any differences in Stabilometric results between patients and HCs were due to age (20). The worse performance of CD patients on the Stroop test as compared to HCs may be attributable to mild impairment in different cognitive domains (21, 22), especially in domains involved in executive functions (23). However, all CD patients had a MoCA score >26, therefore excluding the presence of overt cognitive dysfunction, a condition that would have precluded the execution of the Stroop test.

The novel finding of our study was that CD patients had impaired balance and postural control with increasing difficulties in cognitive tasks. CMI refers to a specific kind of dual-task interference that occurs when the simultaneous performance of a cognitive (i.e. Stroop test) and motor (postural control) task leads to a variable pattern of interference in one or both tasks (24). Dual-task coordination requires the modulation of activities of many specialized information-processing systems. Indeed, several cortical and subcortical structures are likely activated during the maintenance of posture and the execution of the Stroop test. The Stroop test is widely used to measure executive function, although its application is also useful for the evaluation of other cognitive functions, such as attention, processing speed, cognitive flexibility, and working memory (25). The involved brain areas are mainly the anterior cingulate cortex and the dorsolateral prefrontal cortex, which are involved in processing the conflict and resolution processes performed during the test. Postural control and balance are complex biologic functions based on hierarchically-organized procedures that involve the activation of the cortex, brainstem, and medullary structures, which are also influenced by peripheral multisensory information (visual, vestibular, proprioceptive, and somatosensory). The CMI we found in CD patients may be related to impaired connectivity within networks, including connections between cortico-subcortical structures. Although dystonia has been historically viewed as a basal ganglia disorder particularly linked to striatal dysfunction, recent neuroimaging and neurophysiological evidence supports the idea that dystonia is a network disorder (26–28) involving different brain regions, including the basal ganglia, cerebellum, thalamus, and sensorimotor cortex (29). Substantial evidence has demonstrated a widespread impairment in sensory processing and sensorimotor integration in dystonia (30), which specifically involves connections between the sensorimotor cerebral cortex, basal ganglia, and cerebellum. These alterations may contribute to the CMI we found in CD. Furthermore, among the neural circuits involved in cognitive interference in CD patients during a motor task, those responsible for visuospatial processing may also play a role. Indeed, recent studies on CD patients have highlighted the dysfunctional activity of these circuits, including the associative parietal cortex, cerebellum, and subcortical structures (31). Given the high degree of complexity in the brain networks involved in CMI, and considering the pathophysiology of dystonia as a brain network disease (26, 29), it may be hypothesized that during dual-task execution, CD patients have a decreased ability to optimize performance through the recruitment of brain networks or alternative cognitive strategies. In support of this hypothesis, we found a positive correlation between abnormalities in the stabilometric measurements and the number of errors during the Stroop test in CD patients.

The absence of a significant correlation between stabilometric parameters and disease severity, dystonic pattern and the presence of head tremor may suggest that altered CMI does not depend on specific clinical characteristics of dystonia. These results are in line with previous studies reporting no significant changes in stabilometric variables after BONT treatment, which ameliorated head posture (6).

The major limitation of our study is the relatively small sample of CD patients, and therefore our results must be confirmed by future studies conducted on a larger number of patients, including those with abnormal postures not related to a neurological disorder. Furthermore, due to the small sample size, we could not definitively exclude that different CD subtypes had different CMI.

## Conclusions

CD patients showed alterations in CMI mechanisms as compared to age- and sex-matched HCs. These alterations may be linked to altered connectivity of the networks involved in CMI and CD and were not related to patient clinical or demographic data.

Future studies conducted on other forms of focal dystonia and on subjects with other postural abnormalities without a neurological origin as controls are needed in order to strengthen our results and better determine possible therapeutic and rehabilitative interventions aimed at improving the execution of simultaneous motor and cognitive task performance in dystonic patients. Indeed, since CMI has a significant impact on daily life due to an increased risk of falls, it could be a primary target of rehabilitative interventions from disease onset similar to other neurodegenerative disorders (32).

## Data Availability Statement

The raw data supporting the conclusions of this article will be made available by the authors, without undue reservation.

## Ethics Statement

The studies involving human participants were reviewed and approved by Sapienza University of Rome. The patients/participants provided their written informed consent to participate in this study.

## Author Contributions

VB: designed the work, acquisition and interpretation of data, drafted the article and revised it critically, and approved the version to be published. GFe and CC: designed the work, acquisition and interpretation of data, revised the article critically, and approved the version to be published. MD: acquisition and interpretation of data, revised the article critically, and approved the version to be published. DB, GFa, FC, and AC: designed the work, interpretation of data, revised the article critically, and approved the version to be published. MG: designed the work, analysis and interpretation of data, revised the article critically, approved the version to be published. All authors contributed to the article and approved the submitted version.

## Conflict of Interest

The authors declare that the research was conducted in the absence of any commercial or financial relationships that could be construed as a potential conflict of interest.

## References

[1] Jinnah HA Berardelli A Comella C Defazio G Delong MR Factor S. The focal dystonias: current views and challenges for future research. Mov Disord. (2013) 28:926–43. 10.1002/mds.25567 23893450 PMC3733486

[2] Albanese A Bhatia K Bressman SB Delong MR Fahn S Fung VSC. Phenomenology and classification of dystonia: a consensus update. Mov Disord. (2013) 28:863–73. 10.1002/mds.25475 23649720 PMC3729880

[3] Barr C Barnard R Edwards L Lennon S Bradnam L. Impairments of balance, stepping reactions and gait in people with cervical dystonia. Gait Posture. (2017) 55:55–61. 10.1016/j.gaitpost.2017.04.004 28412603

[4] Anastasopoulos D Maurer C Mergner T. Interactions between voluntary head control and neck proprioceptive reflexes in cervical dystonia. Parkinsonism Relat Disord. (2014) 20:1165–70. 10.1016/j.parkreldis.2014.08.008 25175603

[5] De Pauw J Mercelis R Hallemans A Van Gils G Truijen S Cras P. Postural control and the relation with cervical sensorimotor control in patients with idiopathic adult-onset cervical dystonia. Exp Brain Res. (2018) 236:803–11. 10.1007/s00221-018-5174-x 29340715

[6] Moreau MS Cauquil AS Costes Salon MC. Static and dynamic balance function in spasmodic torticollis. Mov Disord. (1999) 14:87–94. 10.1002/1531-8257(199901)14:1<l87::AID-MDS1015>3.0.CO;2-C 9918349

[7] Esposito M Dubbioso R Peluso S Picone A Corrado B Servodio Iammarone C. Cervical dystonia patients display subclinical gait changes. Parkinsonism Relat Disord. (2017) 43:97–100. 10.1016/j.parkreldis.2017.07.005 28712731

[8] Woollacott M Shumway-Cook A. Attention and the control of posture and gait: a review of an emerging area of research. Gait Posture. (2002) 16:1–14. 10.1016/S0966-6362(01)00156-4 12127181

[9] McIsaac TL Fritz NE Quinn L Muratori LM. Cognitive-motor interference in neurodegenerative disease: a narrative review and implications for clinical management. Front Psychol. (2018) 9:2061. 10.3389/fpsyg.2018.02061 30425673 PMC6218850

[10] Raffegeau TE Krehbiel LM Kang N Thijs FJ Altmann LJP Cauraugh JH. A meta-analysis: Parkinson's disease and dual-task walking. Parkinsonism Relat Disord. (2019) 62:28–35. 10.1016/j.parkreldis.2018.12.012 30594454 PMC8487457

[11] Rao AK Uddin J Gillman A Louis ED. Cognitive motor interference during dual-task gait in essential tremor. Gait Posture. (2013) 38:403–9. 10.1016/j.gaitpost.2013.01.006 23369662 PMC3679258

[12] Purcell NL Goldman JG Ouyang B Bernard B O'Keefe JA. The effects of dual-task cognitive interference and environmental challenges on balance in huntington's disease. Mov Disord Clin Pract. (2019) 6:202–12. 10.1002/mdc3.12720 30949551 PMC6417749

[13] Defazio G Albanese A Pellicciari R Scaglione CL Esposito M Morgante F. Expert recommendations for diagnosing cervical, oromandibular, and limb dystonia. Neurol Sci. (2019) 40:89–95. 10.1007/s10072-018-3586-9 30269178

[14] Comella CL Perlmutter JS Jinnah HA Waliczek TA Rosen AR Galpern WR. Clinimetric testing of the comprehensive cervical dystonia rating scale. Mov Disord. (2016) 31:563–9. 10.1002/mds.26534 26971359 PMC4833533

[15] Stroop JR. Studies of interference in serial verbal reactions. J Exp Psychol. (1935) 18:643–62. 10.1037/h0054651

[16] MacLeod CM Dunbar K. Training and Stroop-like interference: evidence for a continuum of automaticity. J Exp Psychol Learn Mem Cogn. (1988) 14:126–35. 10.1037/0278-7393.14.1.126 2963892

[17] Browne J O'Hare N. A quality control procedure for force platforms. Physiol Meas. (2000) 21:515–24. 10.1088/0967-3334/21/4/308 11110249

[18] Lion A Spada RS Bosser G Gauchard GC Anello G Bosco P. “Postural first” principle when balance is challenged in elderly people. Int J Neurosci. (2014) 124:558–66. 10.3109/00207454.2013.864288 24205810

[19] Tjernström F Björklund M Malmström E-M. Romberg ratio in quiet stance posturography–Test to retest reliability. Gait Post. (2015) 42:27–31. 10.1016/j.gaitpost.2014.12.007 25891528

[20] Holtzer R Friedman R Lipton RB Katz M Xue X Verghese J. The relationship between specific cognitive functions and falls in aging. Neuropsychology. (2007) 21:540–8. 10.1037/0894-4105.21.5.540 17784802 PMC3476056

[21] Romano R Bertolino A Gigante A Martino D Livrea P Defazio G. Impaired cognitive functions in adult-onset primary cranial cervical dystonia. Parkinsonism Relat Disord. (2014) 20:162–5. 10.1016/j.parkreldis.2013.10.008 24161376

[22] Conte A Berardelli I Ferrazzano G Pasquini M Berardelli A Fabbrini G. Non-motor symptoms in patients with adult-onset focal dystonia: sensory and psychiatric disturbances. Parkinsonism Relat Disord. (2016) 22(Suppl. 1):S111–4. 10.1016/j.parkreldis.2015.09.001 26360238

[23] Mahajan A Zillgitt A Alshammaa A Patel N Sidiropoulos C LeWitt PA. Cervical dystonia and executive function: a pilot magnetoencephalography study. Brain Sci. (2018) 8:159–65. 10.3390/brainsci8090159 30135369 PMC6162734

[24] Leone C Feys P Moumdjian L D'Amico E Zappia M Patti F. Cognitive-motor dual-task interference: a systematic review of neural correlates. Neurosci Biobehav Rev. (2017) 75:348–60. 10.1016/j.neubiorev.2017.01.010 28104413

[25] Periáñez JA Lubrini G García-Gutiérrez A Ríos-Lago M. Construct validity of the stroop color-word test: influence of speed of visual search, verbal fluency, working memory, cognitive flexibility, and conflict monitoring. Arch Clin Neuropsychol. (2020) 36:99–111. 10.1093/arclin/acaa034 32514527

[26] Jinnah HA Neychev V Hess EJ. The anatomical basis for dystonia: the motor network model. Tremor Other Hyperkinet Mov. (2017) 7:506. 10.5334/tohm.383 29123945 PMC5673689

[27] Conte A Rocchi L Latorre A Belvisi D Rothwell JC Berardelli A. Ten-year reflections on the neurophysiological abnormalities of focal dystonias in humans. Mov Disord. (2019) 34:1616–28. 10.1002/mds.27859 31591783

[28] Conte A Defazio G Mascia M Belvisi D Pantano P Berardelli A. Advances in the pathophysiology of adult-onset focal dystonias: recent neurophysiological and neuroimaging evidence. F1000Res. (2020) 9:F1000. 10.12688/f1000research.21029.1 32047617 PMC6993830

[29] Schirinzi T Sciamanna G Mercuri NB Pisani A. Dystonia as a network disorder: a concept in evolution. Curr Opin Neurol. (2018) 31:498–503. 10.1097/WCO.0000000000000580 29746398

[30] Avanzino L Tinazzi M Ionta S Fiorio M. Sensory-motor integration in focal dystonia. Neuropsychologia. (2015) 79:288–300. 10.1016/j.neuropsychologia.2015.07.008 26164472

[31] Conson M Santangelo G Impallomeni R Silvestre F Peluso S Esposito M. Spatial and egocentric mental rotation in patients with cervical dystonia. J Neurol. (2020) 267:2281–7. 10.1007/s00415-020-09839-8 32307583

[32] Wajda DA Mirelman A Hausdorff JM Sosnoff JJ. Intervention modalities for targeting cognitive-motor interference in individuals with neurodegenerative disease: a systematic review. Exp Rev Neuro. (2017) 17:251–61. 10.1080/14737175.2016.1227704 27548008

